# Thyroid Nodule Molecular Testing: Is It Ready for Prime Time?

**DOI:** 10.3389/fendo.2020.590128

**Published:** 2020-10-09

**Authors:** Tahsin M. Khan, Martha A. Zeiger

**Affiliations:** Surgical Oncology Program, National Cancer Institute, National Institutes of Health, Bethesda, MD, United States

**Keywords:** indeterminate/suspicious thyroid nodule, thyroid cancer, molecular testing, diagnostic testing, management algorithm, thyroid surgery

## Abstract

Cytologically indeterminate thyroid nodules remain a diagnostic and clinical challenge, and molecular testing has been advocated and advanced as a diagnostic modality to help guide treatment. While studies have expounded on the improved diagnostic certainty with these tests, data demonstrating meaningful clinical impact and supporting their routine use is still limited at best. In this review, we discuss the limitations regarding diagnostic accuracy, impact on surgical decision-making and outcomes, and cost-effectiveness of molecular testing. By highlighting the limitations of these tests, we aim to promote more thoughtful utilization of these tools in the management of thyroid nodules going forward.

## Introduction

The incidence of thyroid cancer in the United States is rising exponentially, and thyroid nodules in particular are exceedingly common with prevalence rates of up to 70% in adults (1). Although most thyroid nodules (80–90%) harbor benign pathology, the ability to detect malignancy and determine the appropriate course of treatment is of utmost clinical importance (1). The malignancy potential of a thyroid nodule is discerned in a multimodality fashion that includes clinical history, physical examination, radiographic assessments, and fine needle aspiration (FNA) biopsies (1–5). To this end, cytopathologic analysis of FNAs has emerged as a key adjunct to the clinical and radiologic criteria used to evaluate and characterize thyroid nodules. The Bethesda System for Reporting Thyroid Cytopathology (TBSRTC), developed by the National Cancer Institute in 2009 and revised in 2017, is utilized widely to stratify risk of malignancy based on cytopathology and has been shown to accurately establish diagnosis (benign vs. malignant) in 70–80% of cases (6). Nevertheless, this still leaves 20–30% of FNA cases that fall under the Bethesda III, IV, and V categories of indeterminate or suspicious cytological results. Nodules in these categories have been shown to carry a wide range of malignancy rates ranging from 6 to 75% on final pathology (6). Given this wide variation in results for indeterminate and suspicious thyroid nodules, there has been a concerted effort in developing new technologies to more comprehensively characterize the malignant potential of thyroid nodules.

## Molecular Marker Panels

With recent advances in our understanding of the molecular underpinnings and genotype-phenotype correlations in oncology, molecular testing has become frequently available and implemented in clinical practice for multiple cancer types. The fertile field of thyroid nodules is no exception. For instance, discovery of the oncogenic *BRAF V600E* mutation in high frequency (50–70%) in papillary thyroid cancers and its associated implication for susceptibility to *BRAF* targeted therapies have dramatically shifted the landscape of papillary thyroid cancer treatment (7). In addition to such single gene markers, the past decade has seen the development and marketing of commercial, multi-gene panel molecular tests that aim to improve diagnostic certainty in Bethesda III–IV indeterminate and suspicious nodules. Perhaps the most widely known of these are the Afirma Gene Expression Classified (GEC, since 2011) and ThyroSeq v2 (since 2014), which evaluates mRNA expression levels of 167 genes, or DNA mutations within a 19 gene panel, respectively (8). Both of these tests have been updated since their initial release to Afirma Genomic Sequencing Classifier (GSC, based on RNA sequencing technology evaluating ~10,000 genes) and ThyroSeq v3 (next generation sequencing of 112 genes) (8–10). Additional commercial tests have included assays of oncogenic microRNA expression (e.g., ThyraMIR, Rosetta GX Reveal), and a seven-gene panel test for oncogenic point mutations and gene fusions (11–13). The ongoing evolution of these tests, combined with academic, clinical, and commercial interests, suggests that these molecular tests are now firmly part of the diagnostic armamentarium for thyroid nodules. Indeed, medical societal guidelines have continued to include discussion on molecular testing as diagnostic adjuncts for indeterminate thyroid nodules (1–3, 14–16).

Despite their widespread use, however, do these molecular tests truly impact clinical decision making and subsequent management of patients with indeterminate thyroid nodules? Or are a patient’s history, cytology, and ultrasound findings enough to make sound clinical decisions? Herein we present a critical appraisal of the current state of adjunctive molecular tests used to differentiate benign from malignant nodules and, challenge their true clinical relevance in the context of diagnostic utility, influence on surgical decision-making, and cost effectiveness. We highlight evidence that suggests that the additional diagnostic certainty offered by these tests may be incremental at best from a clinical relevance perspective and that limitations persist pertaining to the real world application and influence on management decision *vis a vis* whether surgical intervention is pursued and if so what operation (17). By summarizing the true clinical impact of these tests and some of the associated pitfalls, we hope to promote future more reasoned and appropriate utilization of these tools in the management of indeterminate thyroid nodules.

## Diagnostic Utility

The Bethesda classification categorizes FNA cytology into six different groups of varying malignant potential (6). Molecular testing is recommended only for “indeterminate or suspicious” nodules classified as “atypia of undetermined significance or follicular lesion of undetermined significance (AUS-FLUS),” “follicular neoplasm or suspicious for a follicular neoplasm (FN-SFN),” or “Suspicious for Malignancy (SFM)” categories. These classifications carry malignancy risk between 5 and 40%, and the primary utilization of molecular testing has centered around obtaining a more definite assessment of this risk (6). Theoretically, any lesion deemed benign by molecular testing would be spared surgery and enter active surveillance pathways. Conversely, lesions deemed malignant would proceed to definitive surgical treatment *via* either lobectomy or total thyroidectomy (1). To this end, molecular tests can be broadly thought of as “rule out” or “rule in” tests for malignancy. The Afirma-GEC is primarily a “rule out” test designed to identify benign biology and thus spare surgery, while the ThyroSeq assay is a “rule in” test that aims to identify nodules that harbor malignancy and thus best treated with surgery (18). However, herein, we aim to carefully examine the real utility of using these molecular panels in the algorithmic approach to thyroid nodule clinical management.

### Afirma

In their seminal paper describing the performance of Afirma-GEC, Alexander and colleagues reported this test to have negative predictive values (NPV) of 95 and 94% for cytologically indeterminate nodules in the AUS-FLUS and FN-SFN categories, respectively (19). These initial excellent numbers suggested that the GEC test could “rule out” malignancy (and thus spare diagnostic surgery) in over 90% of indeterminate nodules, thereby making an substantial case for its implementation into clinical practice. However, subsequent reports have questioned the reproducibility of these results. In particular, later studies have reported that the NPV of Afirma-GEC varied greatly depending on the institution where the FNA biopsies were performed, and in one case was noted to be as low as 69% (7, 20–23). This inter-institutional variation is believed to stem from differences in malignancy prevalence within the evaluated populations. For instance, Marti and colleagues discovered that within the same city, the pre-test malignancy probability of an indeterminate thyroid nodule ranged from 30 to 38% at a cancer center vs. 10–19% for a general hospital, and correspondingly, the NPV for GEC was lower at the cancer center (86–92% vs. 95–98%) (22). Conversely, the positive predictive value (PPV) at the general hospital was too low (14.3 vs. 57.1% at the cancer center), and 86% of the nodules that were resected at that institution based on GEC-suspicious result were ultimately benign on final pathology (22). Furthermore, Al-Qurayshi and colleagues in their extensive analysis of GEC test performance found that this variability could not be accounted for solely by differences in malignancy prevalence, suggesting intrinsic variations in the test’s performance (sensitivity and specificity) (21). The authors highlight that most publications reporting on test performance were at high risk of selection bias given that they were based at single institutions and had relatively small sample sizes (average, 108 nodules). Both impede defining a true sensitivity and specificity value for the test (21). Finally, it has been shown that the GEC does not perform equally across all thyroid nodule histopathology. In particular, studies have demonstrated that samples containing a predominance of Hürthle cells may falsely categorized as being “suspicious” and lead to excessive surgeries in patients with this cytopathology (24, 25). Due to these myriad challenges to its accuracy and NPV, the utilization and interpretation of Afirma test results, in each individual clinical setting, must be thoughtfully considered as it is well known that risk of malignancies, cytology, and pathology results differ across institutions.

### ThyroSeq

Much like the GEC, inter institutional variability has also been observed with ThyroSeq V2. In its initial validation study, this test was reported to have a positive predictive value of 83%; however, this number was found to be drastically lower at 22–43% in a survey of this test’s performance across various institutions by Marcadis and colleagues (26, 27). A similarly low positive predictive value was also reported by Taye and colleague, which casts doubts on this “rule in” test’s ability to accurately identify malignant nodules that require surgical intervention (28). Moreover, both these studies as well as a previous report by Valderrabano and colleagues reported the RAS family of oncogenes to be the most commonly mutated genes in their samples (29). Indeed, although RAS mutations have been demonstrated to be oncogenic drivers in a number of cancer types (such as colon, lung, and pancreas), their role in thyroid malignancy is less well defined. Indeed, in both the Marcadis et al. and Taye et al. studies, the positive predictive value of RAS mutations was found to be between only 7 and 25%, a number that has been replicated by a more recent and in-depth analysis by Guan and colleagues (26, 28, 30). In a systematic review by Najafian et al. RAS mutations have also been shown to be frequently present in benign thyroid nodules, thus further weakening their utility as a oncogenic marker in thyroid cancer (31). Taken together, these results suggest that i) the positive predictive value of ThyroSeq V2 is lower than initially reported and ii) the most common genetic alteration used to establish malignancy by this test may in fact not be a *bona fide* marker of malignant behavior in thyroid cancer.

### Noninvasive Follicular Thyroid Neoplasm With Papillary-Like Nuclear Features and Its Implication

Compounding the challenge to the accuracy of molecular testing is the recent introduction of a new thyroid pathological entity: noninvasive follicular thyroid neoplasm with papillary-like nuclear features (NIFTP). The NIFTP category was created to distinguish an indolent class of encapsulated follicular variant of papillary thyroid carcinoma from other aggressive forms of papillary thyroid cancer. Of note, its average prevalence within indeterminate thyroid nodules is estimated to be 61% (range, 33–81%) (32, 33). Given that these mainstay molecular tests were developed and validated prior to this re-designation (and thus designed to classify this potential benign pathology as malignant), their performance measures have been shown to deteriorate significantly when the NIFTP designation is incorporated in the classification of indeterminate nodules (20, 28, 33–35). For instance, the NPV of Affirma GEC declined from 96 to 81% when the NIFTP designation was retrospectively applied to the cohort studied by Samulski and colleagues (35). Similarly, in the cohort studied by Valderrabano and colleagues, the PPV of ThyroSeq V2 decreased from 42 to 33% when NIFTP lesions were considered benign (29). The performance of the ThyroSeq “rule in” test appears to be more significantly affected by NIFTP reclassification than the Afirma “rule out” test. Nevertheless, the results of both should be interpreted with a consideration that benign NIFTP lesions may be falsely classified as suspicious, and thus subjected to unnecessary surgery by these tests.

### Newer Editions—Afirma-GSC and ThyroSeq V3

Ultimately, the newer versions of these commercial tests appear to be addressing and improving upon these diagnostic limitations. The ThyroSeq V3 test, for instance, has been demonstrated to have a NPV of 97%, and benign call rates of 61% for indeterminate nodules and 53% for nodules with Hürthle cells (9). For the Afirma-GSC, San Martin and colleagues found that the second generation test has a higher specificity (94 vs. 60%) while retaining similar negative predictive value (96.3 vs. 98.6%) compared to the GEC. This resulted in improved performance within specimens with Hürthle cell cytology, improved overall benign call rate of 68% (*vs.* 41%) and a decrease in surgery rate from 48 to 35% for the population as a whole (36). Wei and colleagues have documented that the benign call rate for GSC is considerably higher than GEC (66.7 vs. 45.4%), and Endo and colleagues have reported a benign call rate of 88.8 vs. 25.7% for GSC vs. GEC for samples with Hürthle cells (37, 38). Both these papers, as well as comparative study by Harrell and colleagues, suggested that the rate of surgical intervention was lower during the period when the newer test was utilized (39). However, discrepancies between these initial reports and experience from real world usage are already appearing; Chen and colleagues, for instance, found that the benign call rate of GEC was only 58% compared to the 74% reported in an earlier publication (40, 41). Ultimately, as was learned in the case of Afirma-GEC and ThyroSeq V2, these initial findings and test performances need to be confirmed in both independent validation studies and in larger cohorts. Until then, caution should be urged regarding the application and interpretation of these tests in achieving both diagnostic certainty and truly impacting clinical care in patients with indeterminate thyroid nodules.

## Impact on Surgical Practice

One of the stated aims of molecular testing for indeterminate thyroid nodules is to assess the risk for malignancy, and thus determine 1) whether surgery is appropriate for that lesion and 2), if so, the extent of surgery. A corollary aim to this is reducing the number of diagnostic lobectomies performed, and ultimately spare a patient from the risks and costs of unnecessary surgery. While most studies examining the utility of these molecular tests center on their diagnostic performance (i.e., sensitivity, specificity, NPV, and PPV), investigations on whether implementation of these tests lead to actual changes in surgical utilization and clinical practice patterns are sparse. Prospective studies assessing the impact of these tests on the rate and appropriateness of surgical intervention are lacking, and most of the information is gleaned from retrospective data only. Moreover, many of the series suggesting that implementation of molecular testing leads to reduction in number of surgeries are correlative in nature, i.e., they show that fewer surgeries were done during period of molecular testing use compared to historical time period when testing was not used, but they do not directly show a causal effect at the individual patient/nodule level (36, 39, 42).

### Impact on Surgical Decision Making

One of the first studies to specifically investigate molecular testing’s impact on surgical management was a retrospective study performed by Aragon Han and colleagues in 2014 (43) (**Table 1**). Here, the authors compared management recommendations based on pre-operative molecular testing results to the treatment strategy recommended by a surgical management algorithm. The algorithm was based on clinical parameters developed by experts at a high-volume, tertiary academic institution and in incorporated into a calculator. They found that the strategy influenced by molecular testing differed from the recommendations of the clinical management algorithm in only 10% of the patients (9/87). Furthermore, in 6 out these 9 (67%) patients the molecular testing driven strategy was incorrect and led to overtreatment. Similar results were subsequently observed during two successive investigations by Noureldine and colleagues (44, 45). In one, the authors specifically looked at the appropriateness and impact of Afirma-GEC on management of 273 patients using a similar strategy to Aragon Han et al. and found that the GEC results changed management strategy in just 23 out of 273 patients (8.4%) and led to overtreatment in most of these (72.7%) (44). These results were echoed by a subsequent *prospective* study by the same group where molecular testing only changed management plan in 7.9% patients, out of whom 91% were overtreated (45) (**Table 1**).

**Table 1:** Summary of investigations evaluating impact of molecular testing on surgical management and outcomes.

Series	Study duration	Publication year	Test	Design	Number of patients	Impact of molecular testing
Aragon Han, P. et al. (43)	2009–2013	2014	GEC, Mutation Panel	Retrospective	87	78/87 (90%) no change in management 9/87 (10%) surgical plan altered 6/9 (67%) change inappropriate 5/6 (83%) inappropriately overtreated
Noureldine, S. et al. (44)	2012–2014	2015	GEC	Retrospective	273	250/273 (92%) no change in management 23/273 (8%) surgical plan altered 16/23 (73%) inappropriately overtreated
Marti, J. et al. (22)	2013–2014	2015	GEC	Retrospective	165	At cancer center: 42/70 (60%) GEC suspicious lesions resected 18/42 (43%) GEC suspicious resected lesions ultimately benign (inappropriately overtreated) At general hospital: 21/34 (62%) GEC suspicious lesions resected 18/21 (86%) GEC suspicious resected lesions ultimately benign (inappropriately overtreated)
Noureldine, S. et al. (45)	2014–2015	2016	GEC, ThyroSeq, Mutation Panel	Prospective	140	129/140 (92%) no change in management 11/140 (8%) surgical plan altered 10/11 (91%) inappropriately overtreated
Taye, A. et al. (28)	2014–2016	2018	ThyroSeq	Retrospective	156	37/51 (73%) ThyroSeq suspicious lesions resected 29/37 (78%) ThyroSeq suspicious resected lesions ultimately benign (inappropriately overtreated)

Strikingly, Noureldine and colleagues found that over 50% of the patients who underwent molecular testing did not meet clinical guideline criteria for molecular testing (i.e., already had indications for surgical intervention based on clinical assessment), suggesting that these tests were being inappropriately ordered for a large number of patients whose treatment could be dictated by clinical findings alone. Specifically, clinical parameters alone clearly informed what surgical procedure should be performed. Inappropriate implementation and interpretation of molecular testing was also highlighted by Samulski et al. in their description of GEC utilization at their institution, where they noted that most lesions *not* designated as “Benign” by GEC ultimately underwent surgical resection, even when the sample was classified as “QNS” (quantity not sufficient) and despite the fact that the GEC is a “rule out” test (35). Indeed, 7 out of 13 of these “QNS” lesions in this study were ultimately shown to be benign on final pathology. Taken together, these four studies suggest that 1) molecular testing results influence management strategies in a limited number of patients (<10% of cases), 2) when the strategy does get altered based on molecular testing, there exists a real and significant risk of overdiagnosis and overtreatment, and 3) these tests are often being misused indiscriminately in cases where management decisions can and should be made based on clinical features alone.

Can molecular testing results be used to tailor the extent of surgery for indeterminate nodules? An early investigation by Yip and colleagues appeared to suggest that molecular testing results could guide the decision to offer total thyroidectomy (vs. a diagnostic lobectomy) in lesions with high risk features identified by molecular testing (46). In this study, a positive result in a six gene (BRAF, NRAS, HRAS, KRAS, RET-PTC, and PAX8-PPARy) molecular test panel was used as an indicator for total thyroidectomy for cytologically indeterminate nodule. This approach increased the appropriate use (based on final pathology) of total thyroidectomy by 30% and reduced the rate of lobectomy by 33%, thereby suggesting that molecular testing results could identify malignancy and thus appropriately direct care toward total thyroidectomy while sparing a second operation for completion thyroidectomy in a number of patients. However, a major limitation of this study was incomplete reporting on the rate of false positive outcomes for the molecular test panel (i.e., the number of lesions where unnecessary total thyroidectomy was performed for positive molecular testing result that was ultimately benign on final pathology was not reported). Furthermore, the study was not blinded for the post-operative histopathological assessment of the specimen and, as such, the molecular testing results could have influenced whether an indeterminate lesion was determined as malignant on final pathology, thereby confounding the correlation between pre-operative molecular testing and post-operative pathological assessment. Subsequent investigations into the use of molecular testing, and in particular the commercial tests, in guiding extent of surgery have been limited to case reports [c.f. (47)].

### Implications of New Surgical Guidelines for Thyroid Cancer and NIFTP Diagnosis

Additionally, the utility of molecular testing in guiding the extent of surgery is undermined by changes in clinical guidelines regarding the extent of surgery. The American Thyroid Association, for instance, has suggested that differentiated thyroid cancer <4 cm may be safely treated with lobectomy instead of traditionally performed total thyroidectomy, since limited resection has equivalent outcomes to extensive surgery in select patients (1, 48). Given that fact that more limited cancer operation can lead to equivalent outcomes while sparing patients from important side effects (e.g., need for thyroid hormone replacement after total thyroidectomy), the impact of molecular testing in directing the extent of surgical resection becomes even more diminished.

The advent of NIFTP as a new entity in thyroid nodule classification is another confounding factor undermining the utility of molecular markers in deciding to perform surgery. As discussed above, introduction of the NIFTP terminology to define a more indolent form of thyroid neoplasm significantly affects the diagnostic performance of these molecular tests (33). Additionally, while NIFTP remains a surgically treated entity, the lack of invasive features suggest that a thyroid lobectomy, as opposed to total thyroidectomy, may suffice in its management (49). Given that the commercial molecular tests tend to classify NIFTP lesions as “suspicious,” reliance on these results may lead to overtreatment in the form of total thyroidectomy in lesions that could otherwise be managed by lobectomies. In the face of evolving guidelines and understanding of thyroid cancer biology that now suggests a role for more limited surgical resection, the guidance provided by these molecular markers becomes limited in its scope. Ultimately, it is recommended that the extent of surgery should be determined primarily by clinical variables, ultrasound findings, and individual patient preference (50, 51).

Why does molecular testing, despite the much touted improvement in diagnostic accuracy, fail to have a significant effect on the ultimate decision regarding surgical approach? In addition to the real risk of overdiagnosis and overtreatment summarized above, one hypothesis is that the overall impact of molecular testing on management decision, when compared to that of clinical and radiographic features, is incremental at best. For instance, a recent investigation by Huang and colleagues have suggested that while supplementing clinical information with imaging and cytopathology improved diagnostic accuracy significantly, further incorporation of molecular testing results to these variables led to only a modest and negligible improvement to overall diagnostic capacity (17). Similarly, Vora and colleagues found in their single institution experience that patients with clinical indications for surgery (such as compressive symptoms, interval growth of nodules, and presence of secondary nodules) underwent surgical intervention at high rates despite “benign” calls in the GEC, with 30% of these lesions ultimately being found to harbor malignancy (52). As such, decision-making regarding surgery (be it *whether* to operate or *how much* to resect) remains best determined by clinical factors and the true impact of molecular testing results appear to be limited at best.

## Cost-Effectiveness Analysis

Thyroid surgery, like any invasive procedure, is associated with both direct (e.g., operating room equipment, professional fees, etc.) and indirect costs (e.g., time out of work for the patient, postoperative complications, etc.). It has been postulated that molecular testing can help reduce these costs by more definitively identifying benign nodules and thereby sparing unnecessary surgeries. This claim is put forth in part because despite their own high costs ($4,875 for Afirma-GEC, $6,400 for Afirma-GSC, and $3,200 for Thyroseq V2/3), these molecular tests are cheaper than the $9,000–12,000 cost estimate for a diagnostic lobectomy (13, 53). Additionally, there are a number of studies based on complex modeling of hypothetical clinical scenarios and cost estimates that claim that molecular markers are more cost effective than diagnostic lobectomy (18, 53, 54).

However, similar modeling studies have also suggested that these tests are not cost effective, particularly at their current price points. Najafzadeh and colleagues, for instance, have estimated that the “break even” price at which molecular tests become cost-effective is approximately $1,087, which is significantly lower than the $3,000–6,000 currently being charged for these tests (55). Moreover, a significant limitation of such cost-analysis simulations is that the parameters upon which the models are based (such as assumptions regarding direct costs, diagnostic accuracy, cancer prevalence in the target population, and outcomes after molecular testing vs. thyroid surgery) are typically estimations and averages that in reality can vary greatly amongst institutions. These variables make the findings of these modeling difficult to generalize to real-world situations. Many of the simulations, for instance, have been shown to be sensitive to the direct cost estimates used for modeling (i.e., changing the direct cost variable influences whether molecular testing comes out as cost-effective or not), with some suggesting that the price of these molecular tests need to be lowered in order to achieve cost-effectiveness (18, 56). Finally, in an elegant study incorporating sonographic factors into cost-effectiveness modeling, Zanocco and colleagues noted that molecular testing failed to be cost-effective when lesions already harbored suspicious imaging features (56). This, again, reflected the concept that while attractive in isolation, molecular testing had limited incremental benefit when interpreted in the context of other clinical features in the multidisciplinary evaluation of thyroid nodules. Reports on actual cost accounting after implementation of molecular testing have also suggested limited cost-effectiveness of this technology. A retrospective study reviewing the actual costs incurred after the implementation of molecular testing, for instance, have reported increased costs per patient (57). Applying modeling to real world data obtained retrospectively, Shapiro and colleagues demonstrated that while Afirma utilization could lead to a reduction in the absolute number of patients undergoing surgery (by 13%), its use led to increased overall cost *per nodule* ($2,399 higher compared to no molecular testing over 2 years) given that once classified as “suspicious” on molecular testing these nodules entailed further follow-up interventions (such as repeat biopsy down the road or surveillance imaging) (58). An increase in cost associated with need for ongoing surveillance was also independently demonstrated by Balentine and colleagues who estimated that the lobectomy cost of $6,100 per nodule was significantly less than that for molecular testing (Afirma, $9,400) when calculated over a 5-year period (59). Indeed, Lin and colleagues have estimated that while surgery upfront is more expensive than annual surveillance, this cost difference is reversed by just 16 years of surveillance, which is considerably less than the expected lifespan of these patients (60). Thus, in the long term, surgery may in fact be more cost effective than surveillance for high risk nodules.

## Conclusions and Future Directions

The field of molecular testing for thyroid nodules is expanding rapidly, and incorporation of these tests into the management algorithm for indeterminate thyroid nodules is now advocated by major societal guidelines. While considerable literature exists on the diagnostic performance of commercial molecular tests, there are some major limitations to their application, interpretation, clinical impact, and cost which should be addressed before their routine use (summarized in **Table 2**). Specifically, the variability in diagnostic accuracy posed by variations in test performance and the NIFTP designation, the lack of any true study documenting an impact and appropriate change on surgical management, and the “real world” studies undermining the cost-effectiveness touted by previous modeling studies, all raise concerns regarding the widespread utilization and reliance on these molecular markers. Like any diagnostic test, these expensive investigations are best used in a thoughtful manner and in a case-by-case basis for nodules with equivocal clinical, cytopathologic, and radiographic factors as recommended by professional societies such as the American Thyroid Association and American Association for Endocrine Surgeons. Molecular testing should only be considered if the result of a test would otherwise alter the recommended management of the patient. Indiscriminate and routine use for any suspicious thyroid nodule should certainly be avoided as it could lead to overdiagnosis, increased costs, and most importantly may have no impact on surgical management. Moreover, consideration should also be given to the diagnostic improvement provided by alternate modalities, such as imaging. The American College of Radiology, for instance, has developed the Thyroid Imaging, Reporting, and Data System (TIRADS) algorithm that ascribes malignancy risk to thyroid nodules based on its composition, echogenicity, shape, size, and margins and can provide useful additive information to Bethesda cytology results over that provided by molecular markers (4).

**Table 2:** Advantages and limitations of existing commercial molecular markers.

Category	Consideration
*Diagnostic utility*	*Advantage:* Afirma testing for Bethesda III-IV, if negative and no other indications for surgery, may obviate surgery
	*Disadvantage:* Variability in test performance depending on cancer prevalence rates
	*Disadvantage:* Variability of results depending on underlying cytopathology and histopathology
	*Disadvantage:* Lack of definite correlation between measured biomarker (e.g., RAS mutation) and malignancy
	*Disadvantage:* Introduction of NIFTP diagnosis decreasing risk of malignancy and studies upon which diagnostic marker results determined
*Impact on surgical decision making*	*Advantage:* BRAF and HTERT results may inform adjunctive therapy recommendations
	*Disadvantage:* Limited influence on treatment algorithms otherwise based on clinical and radiographic factors
	*Disadvantage:* Impact on extent of surgery (e.g., lobectomy vs. total thyroidectomy) minimized by changes in surgical guidelines supporting lobectomy for tumors up to 4 cm.
*Cost effectiveness*	*Disadvantage:* Extremely high costs for molecular testing exceeding estimated “breakeven” cost of ~$1,000

Ultimately, given our increasing understanding of thyroid cancer biology, molecular testing needs to become more precise and directed specifically toward informing *what* therapy to offer once thyroid cancer is diagnosed, [such as by the identification of actionable RET, NRTK, BRAF, VEGFR mutations, and pathway alterations that would allow for the use of targeted agents (61–64)]. For instance, there are exciting advanced being made in the research setting on development of new molecular markers (e.g., TERT expression) that can improve prognostication and treatment of thyroid cancer (65). These areas of study are more promising for the treatment of advanced thyroid cancer than what appears to be only incremental changes provided in the diagnosis of indeterminate thyroid nodules. Ultimately, molecular tests should clarify the management algorithm and thus enable us to take more optimal care of patients with thyroid nodules, instead of introducing another layer of information that may be unnecessary and costly.

## Author Contributions

TK and MZ designed, wrote, and edited the manuscript. All authors contributed to the article and approved the submitted version.

## Conflict of Interest

The authors declare that the research was conducted in the absence of any commercial or financial relationships that could be construed as a potential conflict of interest.
